# Supplementary material for: Lactoferrin Prevents Chronic Alcoholic Injury by Regulating Redox Balance and Lipid Metabolism in Female C57BL/6J Mice
Source: Antioxidants (Basel). 2022 Jul 31;11(8):1508. doi: 10.3390/antiox11081508 (PMC9405310; doi:10.3390/antiox11081508)
Supplement: Supplementary file 1 [file antioxidants-11-01508-s001.zip › Supplementary File S1.pdf]

Histological section manufacture

1. Paraffin embedding

1.1 Remove the tissue from the fixed liquid and trim the target tissue with a scalpel in the ventilation cupboard, and put the trimmed tissue and the label in the dehydration box.

1.2 Dehydration and wax leaching: put the dehydration box into the dehydrator in order to dehydrate with gradient alcohol. 75% alcohol 4 hours, 85% alcohol 2 hours, 90% alcohol 2 hours, 95% alcohol 1 hour, anhydrous ethanol I 30 min, anhydrous ethanol II 30 min, alcohol benzene 5~10 min, xylene II 5~10 min,65°C melting paraffin I 1h, 65°C melting paraffin II 1h, 65°C melting paraffin III 1 hour.

1.3 The wax-soaked tissue is embedded in the embedding machine. First, put the melted wax into the embedding frame, and before the wax solidifies, remove the tissue from the dewatering box and put it into the embedding frame according to the requirements of the embedding surface and affix the corresponding label. Cool at-20 °freezing table, and after the wax is solidified, the wax block is removed from the embedded frame and repaired.

2. Paraffin section making

Place the trimmed wax block cool at -20°C freezing table, slice the modified tissue chip wax block on the paraffin slicer, the slice thickness is 4 μm. The tissue is flattened when the slice floats on the 40 warm water of the spreading machine, and the tissue is picked up by the glass slides and baked in the oven at 60°C. After the water-baked dried wax is melted, it is taken out and stored at room temperature.

3. HE staining

3.1 Dewaxing as followed: Xylene I for 20 min; Xylene II for 20 min; 100% ethanol I for 5 min; 100% ethanol II for 5 min; 75% ethanol for 5 min; Rinsing with tap water.

3.2 Stain sections with Hematoxylin solution for 3-5 min, rinse with tap water. Then treat the section with Hematoxylin Differentiation solution, rinse with tap water. Treat the section with Hematoxylin Scott Tap Bluing, rinse with tap water.

3.3 85% ethanol for 5 min; 95% ethanol for 5 min; Finally Stain sections with Eosin

dye for 5 min.

3.4 Dehydrate as followed: 100% ethanol I for 5 min; 100% ethanol II for 5 min; 100% ethanol III for 5 min; Xylene I for 5 min; Xylene II for 5 min; finally seal with neutral gum.

Experimental equipment and reagents

Experimental equipment

**Name**	**Producer**	**Model**
Dehydrator	DIAPATH	Donatello
Embedding machine	Wuhan Junjie Electronics Co., Ltd	JB-P5
Pathology slicer	Shanghai Leica Instrument Co., Ltd	RM2016
Frozen platform	Wuhan Junjie Electronics Co., Ltd	JB-L5
Tissue spreader	Zhejiang Kehua Instrument Co., Ltd	KD-P
Oven	Laibo Rui Instrument Equipment Co., Ltd	GFL-230
Slides	Servicebio	
Dyeing machine	DIAPATH	Giotto

Experimental reagents

**Reagent**	**Producer**	**Code**
Xylene	Sinopharm Group Chemical Reagent Co. LtD	10023418
Ethanol	Sinopharm Group Chemical Reagent Co. LtD	100092683
HE dye solution set	Servicebio	G1003
Neutral gum	SCRC	10004160

Material and method of 16S rDNA sequencing

1. DNA extraction

DNA from the feces was extracted using the E.Z.N.A.® Soil DNA Kit (D4015, Omega, Inc., USA) according to manufacturer's instructions. The reagent which was designed to uncover DNA from trace amounts of sample has been shown to be effective for the preparation of DNA of most bacteria. Nuclear-free water was used for blank. The total DNA was eluted in 50 μL of Elution buffer and stored at -80 °C until measurement in the PCR by LC-Bio Technology Co., Ltd (Hang Zhou, Zhejiang Province, China).

2. PCR amplification and 16S rDNA sequencing

The V3-V4 region of the prokaryotic (bacterial and archaeal) small-subunit (16S) rRNA gene was amplified with primers 341F (5'-CCTACGGGNGGCWGCAG-3') and 805R (5'-GACTACHVGGGTATCTAATCC-3'). The 5' ends of the primers were tagg with specific barcods per sample and sequencing universal primers. PCR amplification was performed in a total volume of 25 μL reaction mixture containing 25 ng of template DNA, 12.5 μL PCR Premix, 2.5 μL of each primer, and PCR-grade water to adjust the volume. The PCR conditions to amplify the prokaryotic 16S fragments consisted of an initial denaturation at 98℃ for 30 seconds; 32cycles of denaturation at 98℃ for 10 seconds, annealing at 54℃ for 30 seconds, and extension at 72℃ for 45 seconds; and then final extension at 72℃ for 10 minutes. The PCR products were confirmed with 2% agarose gel electrophoresis. Throughout the DNA extraction process, ultrapure water, instead of a sample solution, was used to exclude the possibility of false-positive PCR results as a negative control. The PCR products were purified by AMPure XT beads (Beckman Coulter Genomics, Danvers, MA, USA) and quantified by Qubit (Invitrogen, USA). The amplicon pools were prepared for sequencing and the size and quantity of the amplicon library were assessed on Agilent 2100 Bioanalyzer (Agilent, USA) and with the Library Quantification Kit for Illumina (Kapa Biosciences, Woburn, MA, USA), respectively. The libraries were sequenced on NovaSeq PE250 platform.

3. Data analysis

Samples were sequenced on an Illumina NovaSeq platform according to the manufacturer's recommendations, provided by LC-Bio. Paired-end reads was assigned to samples based on their unique barcode and truncated by cutting off the barcode and primer sequence. Paired-end reads were merged using FLAS. Quality filtering on the raw reads were performed under specific filtering conditions to obtain the high-quality clean tags according to the fqtrim (v0.94). Chimeric sequences were filtered using Vsearch software (v2.3.4). After dereplication using DADA2, we obtained feature table and feature sequence. Alpha diversity and beta diversity were calculated by QIIME2, which the same number of sequences were extracted randomly through reducing the number of sequences to the minimum of some samples, and the relative abundance (X bacteria count/total count) is used in bacteria taxonomy. Alpha diversity and Beta diversity were analyzed by QIIME2 process, and pictures were drawn by R (v3.5.2). The sequence alignment of species annotation was performed by QIIME2 plugin feature-classifier, and the alignment database was SILVA and NT-16S.
