# Supplementary material for: Lactoferrin Prevents Chronic Alcoholic Injury by Regulating Redox Balance and Lipid Metabolism in Female C57BL/6J Mice
Source: Antioxidants (Basel). 2022 Jul 31;11(8):1508. doi: 10.3390/antiox11081508 (PMC9405310; doi:10.3390/antiox11081508)
Supplement: Supplementary file 1 [file antioxidants-11-01508-s001.zip › Supplementary Table S1.pdf]

Supplementary Table S1 Ingredients of the diets

ingredient (g)	CON & EtOH	LLf	HLf
casein	200	199.2	192
lactoferrin	0	0.8	8
L-cystine	3	3	3
sucrose	100	100	100
cornstarch	397.5	397.5	397.5
dyetrose	132	132	132
soybean oil	70	70	70
cellulose	50	50	50
mineral mix #210025	35	35	35
vitamin mix #310025	10	10	10
choline bitartrate	2.5	2.5	2.5
total	1000	1000	1000
kcal/g	4	4	4

CON, control group; EtOH, ethanol intervention group; LLf, low-dose lactoferrin group; HLf, high-dose lactoferrin group.
