# Supplementary figures and images for: Lactoferrin Prevents Chronic Alcoholic Injury by Regulating Redox Balance and Lipid Metabolism in Female C57BL/6J Mice
Source: Antioxidants (Basel). 2022 Jul 31;11(8):1508. doi: 10.3390/antiox11081508 (PMC9405310; doi:10.3390/antiox11081508)

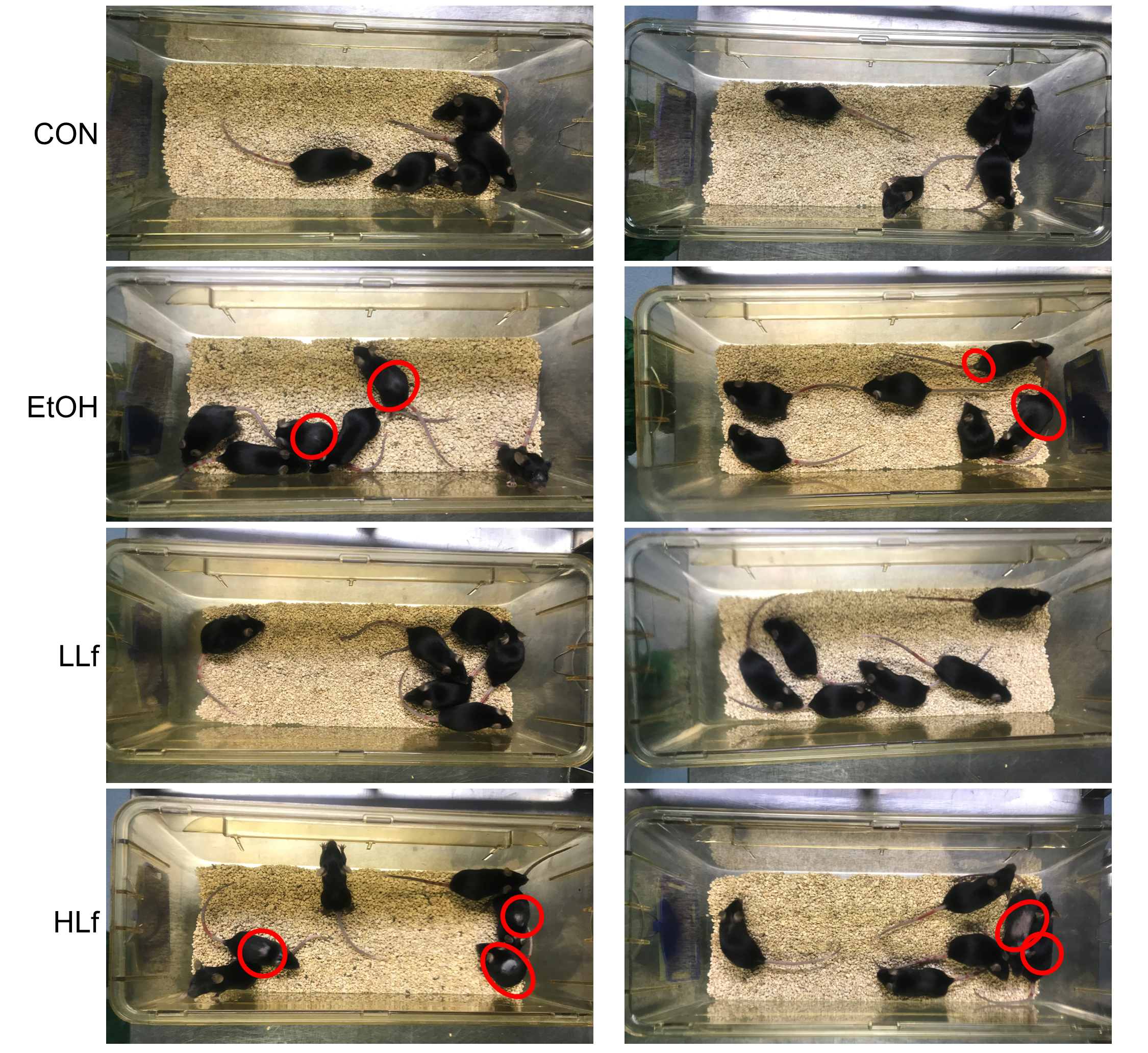

Supplement: Supplementary file 1 [file antioxidants-11-01508-s001.zip › Supplementary Figure S1.tif]
